# The Influence of *Juglans regia* L. Extract and Ellagic Acid on Oxidative Stress, Inflammation, and Bone Regeneration Biomarkers

**DOI:** 10.3390/ijms252312577

**Published:** 2024-11-22

**Authors:** Alina Hanga-Farcas, Luminita Fritea, Gabriela Adriana Filip, Simona Clichici, Laura Gratiela Vicas, Vlad-Alexandru Toma, Eleonora Marian, Felicia Gabriela Gligor, Wael Abu Dayyih, Mariana Eugenia Muresan

**Affiliations:** 1 Doctoral School of Biomedical Sciences, University of Oradea, 1 University Street, 410087 Oradea, Romania; alina.hanga@didactic.uoradea.ro; 2 Department of Preclinical Discipline, Faculty of Medicine and Pharmacy, University of Oradea, 10, 1 December Square, 410073 Oradea, Romania; lfritea@uoradea.ro (L.F.); mmuresan@auoradea.ro (M.E.M.); 3 Department of Physiology, Iuliu Hatieganu University of Medicine and Pharmacy, 8 Victor Babes Street, 400347 Cluj-Napoca, Romania; sclichici@umfcluj.ro; 4 Department of Pharmacy, Faculty of Medicine and Pharmacy, University of Oradea, 10, 1 December Square, 410073 Oradea, Romania; lvicas@uoradea.ro (L.G.V.); emarian@uoradea.ro (E.M.); 5 Department of Molecular Biology and Biotechnology, Faculty of Biology and Geology, Babes-Bolyai University, 5–7 Clinicilor Street, 400006 Cluj-Napoca, Romania; vlad.toma@ubbcluj.ro; 6 Faculty of Medicine, Lucian Blaga University Sibiu, Lucian Blaga Street, No 2A, 550169 Sibiu, Romania; feligligor@yahoo.fr; 7 Department of Pharmaceutical Chemistry, Faculty of Pharmacy, Mutah University, Al Karak 61710, Jordan; wabudayyih@mutah.edu.jo

**Keywords:** *Juglans regia* L. extract, ellagic acid, malondialdehyde, superoxide dismutase, catalase, tumor necrosis factor-α, interleukin-6, RANKL, hydroxyproline, bone regeneration

## Abstract

Bone regeneration is a highly dynamic and complex process that involves hematopoietic stem cells and mesenchymal cells, collagen fibers, non-collagenous proteins and biomolecules from extracellular matrices, and different cytokines and immune cells, as well as growth factors and hormones. Some phytochemicals due to antioxidant and anti-inflammatory effects can modulate the bone signaling pathways and improve bone healing and thus can be a good candidate for osteoregeneration. The aim of this study was to analyze the impact of *Juglans regia* L. extract compared to ellagic acid on bone neoformation in rats. The animals with a 5 mm calvaria defect were divided into four groups (n = 10): group 1 was treated with ellagic acid 1% (EA), group 2 was treated with *Juglans regia* L. extract 10% (JR), group 3 was treated with a biphasic mix of hydroxyapatite and tricalcium phosphate (Ceraform), and group 4 was treated with vehicle inert gel with carboxymethylcellulose (CMC). After 3 weeks of treatment, blood samples were collected for oxidative stress and inflammation assessment. Additionally, the receptor activator of nuclear factor kappa-Β ligand (RANKL) and hydroxyproline levels were quantified in blood. The skull samples were analyzed by scanning electron microscopy in order to detect the modifications in the four groups. The results suggested that JR extract had relevant anti-oxidant effect and bone protective activity and generated the accumulation of Ca and P, demonstrating the potential therapeutic abilities in bone regeneration.

## 1. Introduction

In orthopedic clinical practice, you have to deal with different bone defects that often lead to a decrease in the quality of life of our patients, with considerable financial costs regarding the treatment of these pathologies. Moreover, the treatment of bone defects still represents a major clinical problem that can be a complication of trauma, infections, or degenerative or tumoral pathologies. Their treatment consists of the complete removal of the affected bone tissue and the implantation of a material that replaces the tissue but at the same time stimulates its regeneration.

Currently, in medical practice for the treatment of bone defects allografts, autografts, metal or ceramic implants, demineralized bone matrix or growth factors are used but all of these have many disadvantages. These materials must have several properties: biocompatibility, osteoconductivity, osteoinductivity, nontoxicity, and physical properties comparable and compatible with those of bone tissue. That’s why finding materials with properties similar to bone tissue is a constant challenge for the practitioner.

It is known that bone regeneration is coordinated by two processes: bone resorption by osteoclasts originating from hematopoietic cells [1] and the formation of new bone tissue by osteoblasts, cells derived from mesenchymal cells [2]. The two processes are in balance and are coordinated particularly by osteocytes, mature bone cells located in the bone matrix [3].

Bone healing is a complex mechanism of bone regeneration that takes place in four phases. Inflammation, the first phase, occurs immediately after the lesion at the site of the injury. The blood vessels break, and the formation of a hematoma occurs, which represents a source of hematopoietic cells capable of releasing pro-inflammatory growth factors such as tumor necrosis factor-alpha (TNF-α), bone morphogenetic proteins (BMP), and interleukins (IL-1, IL-6, IL-11, IL-23). These molecules attract macrophages and monocytes, which phagocytose the dead bone cells and secrete vascular endothelial growth factor (VEGF) to stimulate angiogenesis and healing. In the second phase under the control of BMPs, mesenchymal stem cells (MSCs) migrate to the lesion site and differentiate into fibroblasts, osteoblasts, and chondroblasts; as a result, chondrogenesis occurs with the formation of a fibrocartilaginous callus (soft callus). In the third phase, the soft callus undergoes endochondral ossification, thus the fibrocartilaginous callus turns into bone callus (hard callus), while in a final stage that can last for years, bone remodeling occurs under the action of osteoclasts [4].

Bone regeneration and bone healing are modulated by a series of hormones (Parathormon), biomarkers (Osteopontin, Osteocalcin, RANKL), multiple signaling pathways, and transcriptional factors (receptor activator of nuclear factor κappa-B—RANK/RANKL/osteoprotegerin—OPG, NOTCH, Wnt). Moreover, the imbalance between reactive oxygen species (ROS) and the activity of antioxidants leads to oxidative stress [5], a process involved in normal homeostasis of bones. When it becomes excessive, it can delay bone healing, result in the appearance of inflammation, and apoptosis of tissue.

Various plant extracts have been effective in improving bone regeneration due to their composition in several phytocompounds with demonstrated potential in orthopedic applications by estrogenic activity, antioxidant and anti-inflammatory properties, and bone pathway modulation [6]. Thus, polyphenols from *Dalbergia sissoo* increased bone volume fraction and mineralization and induced the expression of genes involved in osteogenesis such as BMP-2, BMP-4, RunX-2, and COL-1 [7].

Based on these mechanisms and due to the numerous disadvantages of the classic regeneration methods, the current study aims to highlight the beneficial qualities of walnut (*Juglans regia*) leaf extract on bone tissue. Known for its anti-inflammatory and antioxidant effects, walnut leaf extract is insufficiently studied as a possible osteoregenerative treatment. *Juglans regia* L., or walnut, belongs to the Juglandaceae family, and extracts prepared from fruits, roots, stems, and leaves have been shown to have antioxidant and anti-inflammatory effects. On bone metabolism, *Juglans regia* L. had a positive effect on the expression of osteogenic genes in human bone marrow mesenchymal stem cells, promoting cell differentiation through BMP2/small mother against decapentaplegic (Smad)/Runx-related transcription factor 2 (Runx2) and Wnt/β-catenin signaling pathways [8].

Ellagic acid is a polyphenol naturally found in extracts of hazelnuts and other plants with important antioxidant, anti-inflammatory, and anti-hepatotoxic properties. Previous studies have also demonstrated its ability to inhibit cancer cell proliferation and induce cell apoptosis [9]. Known for his pro-osteogenic effects, ellagic acid inhibits the activation of nuclear factor kappa B (NF-kB) and interleukins (IL-10 and IL-4), as well reducing the expression of pro-inflammatory cytokines, such as interleukin 1p (IL-1p), tumor necrosis factor (TNF), and interleukin 6 (IL-6) [10]. Recent studies have demonstrated the ability of ellagic acid to block the binding of RANKL to RANK and to inhibit osteoclastogenesis [11].

Starting from these findings, the aim of the study was to evaluate the ability of *Juglans regia* extract compared to ellagic acid to heal the bone tissue and their interaction with biomarkers of oxidative stress, inflammation, and bone regeneration.

## 2. Results

### 2.1. Characterization by HPLC-UV Analysis of the Extract and Evaluation of Antioxidant Activity

The determination of total phenolic compounds from the walnut extract found values between 878.15 µg/g and 0.34 µg/g dry weight (d.w.). Quantities higher than 300 µg/g d.w. were registered for caffeic acid (878.15 ± 1.50 µg/g d.w.), quercetin (328.11 ± 1.39 µg/g d.w.), and (+)- catechin (322.01 ± 1.49 µg/g d.w.). Values lower than 1 µg/g d.w. were recorded for ellagic acid (0.34 ± 0.02 µg/g d.w.) and cinnamic acid (0.50 ± 0.02 µg/g d.w.) (Table 1).

The total polyphenolic and flavonoid content, as well as the antioxidant activity, were also assessed for the JR extract. Total polyphenol content was 144.96 mg GAE/g DW and flavonoid content was 102.74 ± 10.02 mg QE/g DW. Using a DPPH reagent, we calculated the antioxidant capacity of the walnut extract, and obtained a value of IC_50_ of 30.69 µg/mL of extract (0.03 mg/mL extract).

The phytochemical characterization of the extract revealed the presence of 10 phenolic acids and flavonoids, some of them found in considerable concentrations, leading to significant antioxidant activity.

### 2.2. Oxidative Stress Biomarkers, Cytokines, and Matrix Protein Levels

**Figure 1:**
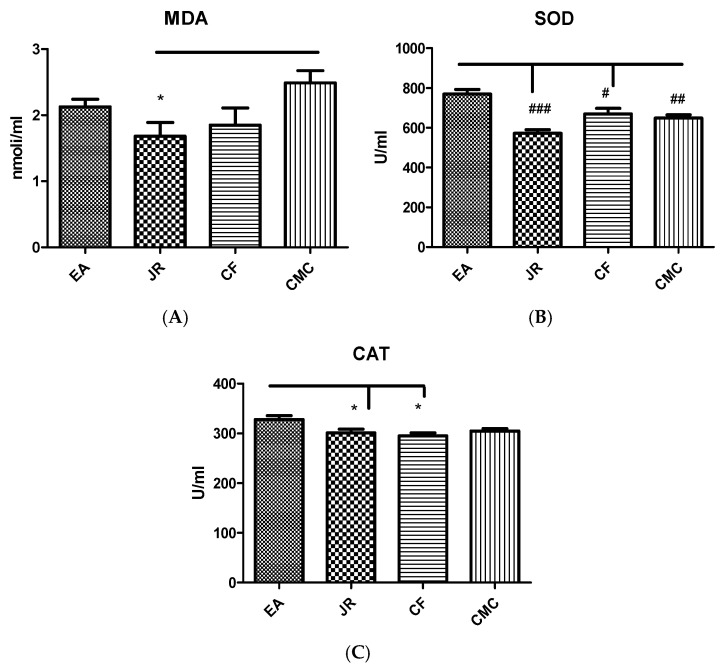
MDA levels (**A**), and SOD (**B**) and CAT (**C**) activities in blood in the experimental groups treated with EA, JR, and CF extract compared to the CMC group. JR extract reduced the MDA levels (*p* < 0.05) compared to CMC group and maintained a diminished SOD and CAT activities in blood (*p* < 0.001 and *p* < 0.05) compared to EA. CF did not improve the oxidative stress parameters and preserved the reduced activity of SOD and CAT (*p* < 0.05) compared to EA. * *p* < 0.05 vs. CMC group; # *p* < 0.05; ## *p* < 0.01; and ### *p* < 0.001 vs. EA group.

The quantification of oxidative stress biomarkers, such as MDA levels, and SOD and CAT activities was assessed from the blood (Figure 1).

MDA is used as an indicator of lipid peroxidation. The MDA values for the group treated with JR were quantified within 1.68 ± 0.54 nmol/mL in comparison with the group CMC (2.48 ± 0.52 nmol/mL) (Figure 1A). MDA levels after JR administration decreased significantly compared to the CMC group (*p* < 0.05). In the groups treated with EA (2.12 ± 0.30 nmol/mL and CF (1.84 ± 0.63 nmol/mL, there were no significant differences (*p* > 0.05) vs. CMC.

The SOD activity was recorded within 769.3 ± 62.50 U/mL for the group that received EA, 572.2 ± 48.91 U/mL for JR, and 669.8 ± 68.55 U/mL for CF. Regarding the group with CMC, the values were 648.5 ± 48.24 U/mL. (Figure 1B). SOD activity in the experimental group treated with EA was significantly increased compared to all other groups (*p* < 0.05) and decreased significantly in the group treated with JR compared to the CF group (*p* < 0.05).

CAT activity was registered as results 32.7 ± 21.60 U/mL for EA, 301.2 ± 19.00 U/mL for JR, and 295.1 ± 14.86 U/mL for CF, and 304.8 ± 13.74 U/mL for CMC(Figure 1C). CAT achieved a slight decrease in activity in both JR- and CF-treated experimental groups. The CAT activity as a parameter for evaluation of antioxidant status in the blood of animals enhanced significantly after treatment with EA in comparison to those treated with JR and CF (*p* < 0.05).

**Figure 2:**
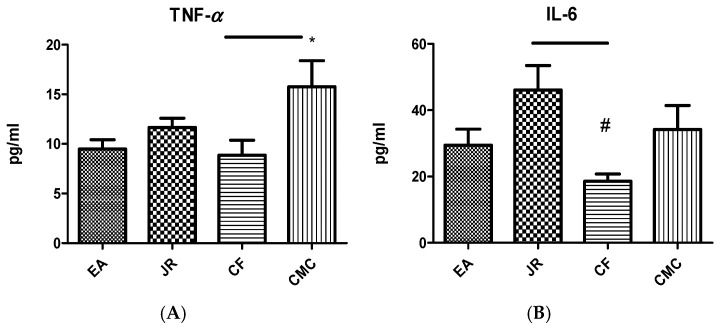
TNF-α (**A**) and IL-6 (**B**) values in serum in the experimental groups treated with EA, JR, and CF extract compared to the CMC group. EA, JR extract, and CF reduced the TNF-α levels (*p* < 0.05) compared to CMC group. EA and CF reduced the IL-6 levels (*p* < 0.05) compared to CMC group. * *p* < 0.05 vs. CMC group; # *p* < 0.05 vs. CF group.

The protective effect of the JR on induced inflammation was studied by evaluating the TNF-α and IL-6 levels in blood (Figure 2).

TNF-α values had no significant effect on the group treated with EA (9.47 ± 2.49 pg/mL) compared to JR (11.66 ± 2.47 pg/mL) and CF (8.86 ± 4.28 pg/mL) (Figure 2B). The treatment with EA and JR of the experimental groups showed no significant modification in IL-6 and TNF-α secretion. The administration of the CF diminished the IL-6 level compared to the CMC group (*p* < 0.05).

The IL-6 values had no significant modification for EA administration (29.47 ± 12.66 pg/mL) vs. JR (46.08 ± 19.56 pg/mL), vs. CF (18.60 ± 5.33 pg/mL), and vs. CMC (34.17 ± 19.18 pg/mL) (Figure 2A). IL-6 increased significantly in the group treated with JR compared to the CF group (*p* < 0.05).

**Figure 3:**
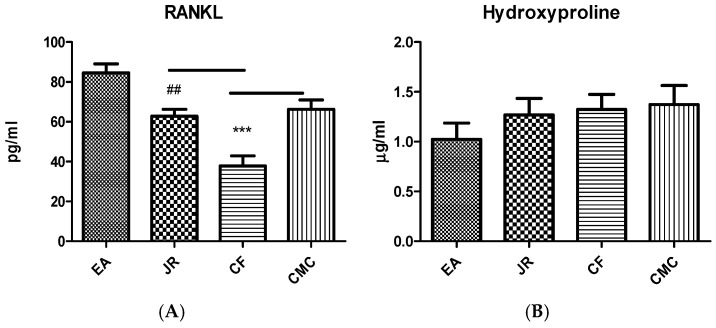
RANKL (**A**) and Hydroxyproline (**B**) values in serum in the experimental groups treated with EA, JR, and CF extract compared to the CMC group. JR extract and CF reduced the RANKL levels (*** *p* < 0.001) compared to CMC group, meanwhile EA increased it. JR extract increased the RANKL levels compared to CF group (## *p* < 0.01). EA, JR extract and CF did not change significantly the hydroxyproline levels compared to the CMC group.

Proinflammatory cytokines stimulate the expression of RANKL, a bone marker that belongs to the TNF family and is essential for osteoclast formation. RANKL values (Figure 3A) increased significantly in the group treated with EA (84.50 ± 11.99 pg/mL), compared to the JR (62.80 ± 9.30 pg/mL), CF (37.81 ± 12.46 pg/mL), and CMC (66.33 ± 13.06 pg/mL) groups (*p* < 0.05). RANKL production also increased significantly in the group treated with JR compared to the CF group (*p* < 0.05) and diminished significantly in the group treated with CF compared to the CMC group (*p* < 0.05).

Hydroxyproline is regarded as a marker of bone reabsorption. In this study, blood level of hydroxyproline among the groups treated with EA (1.02 ± 0.43 µg/mL), JR (1.26 ± 0.43 µg/mL), and CF (1.32 ± 0.36 µg/mL) had no changes compared to the control group (1.37 ± 0.54 µg/mL) (*p* > 0.05) (Figure 3B).

Therefore, the oxidative stress was diminished in the presence of JR extract (MDA levels were lowest in the JR group), while the proinflammatory mediators were not significantly modified. Concerning the biomarkers for bone regeneration and reabsorption, the RANKL levels were low in the JR group, and hydroxyproline concentration was not significantly improved.

### 2.3. SEM Analysis

**Figure 4:**
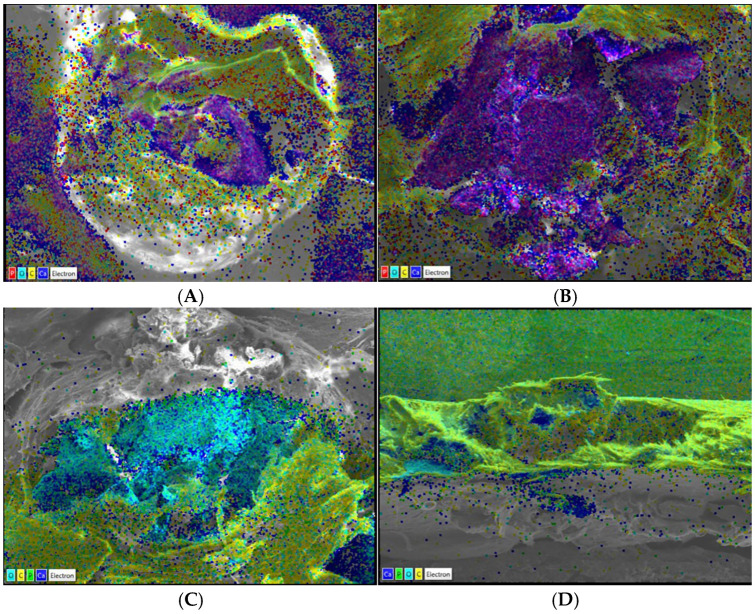
Localized spectroscopy analysis in placebo and treated rats: (**A**) placebo, (**B**) ceraform, (**C**) ellagic acid 1%, and (**D**) Walnut leaf extract 10%.

The skull samples from the four groups were also analyzed by SEM (Figure 4).

The localized spectroscopy analysis showed that the 1% EA therapy tends to increase the signal of calcium (blue) and oxygen (turquoise) (Figure 4C), compared to the action of the JR extract that leads the regenerative process towards the accumulation of phosphorus (green) and carbon (yellow) (Figure 4D). The standard therapy with CF (Figure 4B) induced a prominent calcium accumulation in the lesional area compared to placebo (Figure 4A) and the other treated groups. In the JR group, the phosphorus was accumulated, being an important component of hydroxyapatite involved in bone biomineralization.

## 3. Material and Methods

### 3.1. Preparation and Characterization of Juglans regia L. Extract

The dried leaves of *Juglans regia* L. were purchased from a local tea company (Fares, Orăștie, Hunedoara, Romania). The plant material was ground to a fine powder with an electric grinder. The extraction was performed according to the specifications from the Romanian and European Pharmacopoeia [12,13]. A total of 5.00 g of plant material (*Juglans folium*) were placed in a Berzelius beaker, and 50 mL of 70% (*v*/*v*) alcohol was added. The contents of the beaker were kept at room temperature for 10 days and stirred periodically. After the extraction was completed, the extractive solution was separated and filtered. Next, the fluid extract was subjected to evaporation in a hot air oven (Pol Eko model SLW 115, Wodzislaw Slaski, Poland) to dryness at 45 °C to remove the volatile fraction. The yield obtained was 86%.

### 3.2. High-Performance Liquid Chromatography Ultra-Violet (HPLC-UV) Analysis

The HPLC-UV analysis for the identification and quantification of several phenolic compounds was carried out by using a Shimadzu SCL-40 HPLC system (Kyoto, Japan) equipped with a degasser, quaternary pump, photodiode array detector, thermostatted column oven, and autosampler. The column used was Nucleosil C18 (250 mm × 4.6 mm, i.d. 5 µm) (Sigma-Aldrich, Merck Group, St. Louis, MO, USA). The oven temperature was 25 °C. The elution was performed by using three mobile phases: A, purified water; B, methanol; and C, 96:4 (*v*/*v*) purified water–acetic acid in a gradient program: 15% B and 85% C at 0 min, 75% A and 25% B at 15 min, 15% A and 85% B at 20 min, 40% A and 60% B at 40 min, followed by column conditioning. The flow rate was 0.5 mL/min for the first 15 min and 0.8 mL/min from minute 15. The detection was performed at 280 nm for gallic acid, (+)-catechin, syringic acid, and cinnamic acid, 306 nm for resveratrol, 330 nm for caffeic acid and ferulic acid, and 360 nm for rutin and quercetin; the injection volume was 250 µL [14]. The standards of gallic acid (purity > 99%), ferulic acid (purity > 99%), syringic acid (purity > 95%), cinnamic acid (purity > 99%), caffeic acid (purity > 99%), (+)-catechin (purity > 98%), resveratrol (purity > 99%), quercetin (purity > 95%), and rutin (purity > 94%) were purchased from Sigma-Aldrich (Merck Group, St. Louis, MO, USA).

For the identification and quantification of the ellagic acid, the analysis was carried out by using a method adapted from the one proposed by An et al. [15]. The column used was Nucleosil C18 (250 mm × 4.6 mm, i.d. 5 µm). The oven temperature was 30 °C. The elution was performed in isocratic mode by using a mobile phase consisting of 80% purified water and 20% acetonitrile at a flow rate of 1 mL/min for 10 min, followed by column conditioning. The injection volume was 1 µL, and the detection was performed at 254 nm [13]. The standard of ellagic acid (purity > 99%) was purchased from Sigma-Aldrich.

### 3.3. Evaluation of Total Polyphenolic Content

The technique used for determining the total polyphenolic content was the Folin–Ciocâlteu method, described by Jurca et al. [16]. The assessment was carried out in triplicate, and results were expressed as mean value ± DS, as mg gallic acid equivalent (GAE) (mg GAE/100 g DW).

### 3.4. Determination of Flavonoid Content

The total flavonoid content was measured using the colorimetric method. The absorbance was measured at 510 nm, using an ultra-violet–visible spectroscopy (UV-VIS) PG Instruments T70+ spectrophotometer (Leicestershire, UK), and the results were expressed as mg quercetin equivalents (QE)/g DW. The equation of the calibration curve was: 56.818571x − 0.066498 (R^2^ = 0.9983), where y = absorbance, and x = concentration in mg QE/mL [16]. The evaluation was performed in triplicate, and the results were expressed as mean value ± DS, as mg QE/g DW.

### 3.5. Assessment of Antioxidant Capacity Using 2,2-Diphenyl-2-Picryl-Hydrazylhydrate (DPPH) Reagent

A DPPH reagent was used based on the method described by Jurca et al. [16]. The determinations were performed in triplicate and the results were expressed as means ± DS.

### 3.6. In Vivo Study

For in vivo treatment, JR extract and EA were incorporated into 2% CMC mucilage at a concentration of 10% for JR and 1% for EA. Ceraform (CF), a synthetic bone substitute (produced by Teknimed, Vic en Bigorre, France), is a ceramic manufactured from a biphasic mix of hydroxyapatite and tricalcium phosphate and was used as a positive control. Negative control was treated with vehicle, inert gel of CMC.

**Figure 5:**
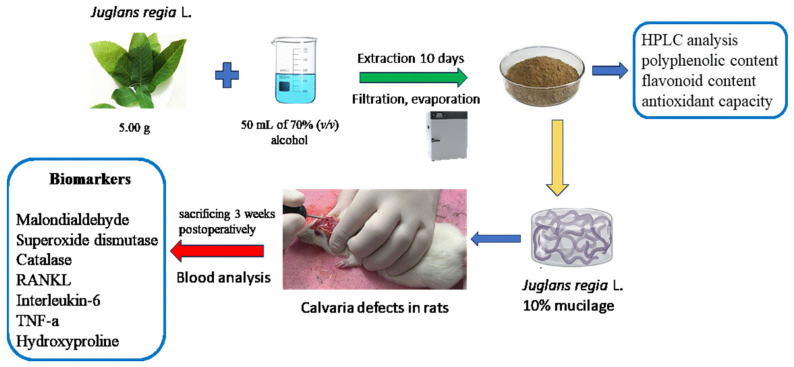
Experimental design.

The in vivo study was performed on 36 female Wistar rats with bone defects realized in calvaria. The 3-month-old animals, weighing 150 ± 10 g, were obtained from the University of Medicine and Pharmacy Cluj-Napoca biobase and were divided into four equal groups (n = 9). The experimental design is presented in Figure 5. The animals were operated on under general anesthesia with ketalnar 40 mg/kg intraperitoneally. A 2 cm sagittal incision on the head midline was performed. Two small bony defects [17], each of 5 mm, considered to be a critical size defect [18], were made at the calvarian bone on either side of the sagittal suture by drilling, under a continuous saline solution flush, to prevent tissue burn and the traumatization of the dura mater and the sagittal suture. Each defect was filled with 0.1 mL of EA 1% for group 1, JR extract 10% for group 2, CF for group 3, and CMC inert gel for group 4. After the treatment, a collagen membrane (Evolution OsteoBiol, Tecnoss, Giaveno, Italy) [19] of 5 mm was applied to prevent the substance diffusion at the soft tissue level, and the tegument was sutured.

For a period of three weeks preoperatively, the animals were acclimatized, being kept in clean cages with access to food and water at a temperature of 20–24 °C with a humidity of 40–60% in a cycle of 12 h light–12 h dark, and the same conditions persisting postoperatively as well.

The animals were sacrificed 3 weeks postoperatively for the collection of blood for biochemical analyses.

### 3.7. Oxidative Stress Assessment

The redox imbalance was quantified by measurement of malondialdehyde (MDA) as a marker of oxidative stress and activity of superoxide dismutase (SOD) and catalase (CAT) as markers of endogenous antioxidant activity. The results were expressed as nmoles/mL for MDA and U/g protein for SOD and CAT activities [20].

### 3.8. Evaluation of Cytokines and Matrix Protein Levels

RANKL, IL-6, TNF-α, and hydroxyproline levels were evaluated by the enzyme-linked immunosorbent assay (ELISA) technique in the blood samples, and the results were expressed in pg/mL.

### 3.9. Scanning Electron Microscopy (SEM) Analysis

A bone trepanation lesion was induced in Wistar rats to obtain a hole in the skull. The procedure involved drilling away a portion of the skull bone to access the underlying brain tissue. The purpose of trepanation was to evaluate the bone response after the lesion in terms of chemical elements detected by energy dispersive spectroscopy (EDS) scanning microscopy.

Preparation of fragment samples from the cranial cap, in which two perforations were made for scanning electron microscopy, was performed as follows: the samples were co-preserved in formol, and in the first step, 2 washes were performed in ultrapure water, after which the samples were left to dry for 12 h. A fragment of 1 square cm was cut from each sample at the level of the area where the bone lesions were created. After dehydration, the samples were fixed individually on a brass support (strap) for the microscope with the help of self-adhesive carbon sticks of 12 mm diameter and silver paste (conductive silver paint from Agar Scientific (Rotherham, UK). After mounting on the supports, the samples were metallized in the Agar Sputter Coater in an argon atmosphere with a 10 nm layer of platinum to obtain conductivity, which is essential for obtaining quality images. Images were obtained using the Hitachi SU8230 scanning electron microscope (Hitachi High-Tech, Hitachi, Japan). The images were obtained at an electron acceleration of 30 kV [21].

## 4. Discussions

The study evaluated the JR extract on bone healing in rats with calvaria defects compared to ellagic acid. JR extract demonstrated antioxidant properties and diminished RANKL secretion without influencing hydroxyproline levels. Additionally, JR extract induced the accumulation of phosphorus and carbon, while CF diminished the inflammation and RANKL levels without improvement of antioxidant capacity.

JR extract was used due to the high content of polyphenols and demonstrated good antioxidant capacity. These properties of JR extract are in agreement with the literature data, but obviously, it could depend on environmental factors, soil composition, and the maturity level of leaves [22,23,24,25]. A physicochemical analysis/characterization of the studied JR extract was carried out to confirm the presence of flavonoids and phenolic acids (including EA), as well as the antioxidant activity.

Thus, by HPLC analysis, a number of 10 phenolic acids and flavonoids were quantitatively determined; in the studied extract were found higher amounts of caffeic acid (878.15 ± 1.50 µg/g d.w.), ferulic acid (218.55 ± 1.42 µg/g d.w.), catechin (322.01 ± 1.49 µg/g d.w.), and quercetin (328.14 ± 1.39 µg/g d.w.). Total polyphenol content and flavonoid content were also assessed, obtaining 144.96 mg GAE/g DW and 102.74 ± 10.02 mg QE/g DW, respectively. Concerning the antioxidant capacity of the walnut extract, an IC50 value of 30.69 µg/mL extract was obtained (0.03 mg/mL extract).

These compounds have demonstrated efficacy in bone regeneration. Thus, a number of studies have shown the beneficial roles of quercetin in bone regeneration, such as Wong et al.’s study [26]. Other studies have demonstrated the osteoprotective role of catechin from green tea extract [27]. Caffeic acid proved its effectiveness in an in vivo experiment in bone healing in rats [28], while ferulic acid demonstrated osteoregenerative properties in a study performed by Liang et al. on an irradiated bone defect [29].

Recent studies had demonstrated that the use of JR extract, due to their contained bioactive compounds, reduced the level of ROS and also had anti-inflammatory and antiproliferative properties and could increase the activity of osteoblasts with a beneficial effect on bone loss [30,31,32,33]. Thus, Pang et al. investigated the effects of JR leaf extract on the osteogenesis of human bone marrow mesenchymal stem cells, demonstrating that it regulates the osteogenic differentiation and cellular autophagy of human bone marrow mesenchymal stem cells (hBM-SCs) through the BMP2/Smad/Runx2 and Wnt/β-catenina [8].

In a study by Zhou et al., the antioxidant activity of defatted walnut kernel extract and whole walnut kernel extract was evaluated in vitro and in vivo. The in vitro results showed that both extracts had antioxidant activity and reduced oxidative stress in mice [34]. According to Calcabrini et al., the extracts from *Juglans regia* L., due to their bioactive constituents, were reported to inhibit the production of ROS [30]. The antioxidant effect (by reducing ROS and NO generation), anti-inflammatory, neuroprotective, and antiproliferative effects of EA, a bioactive compound from the JR extract, have been demonstrated in age-related neurological disorders [35]. A walnut-derived peptide significantly increased CAT and SOD activities, while decreasing MDA concentration both in vitro and in vivo experiments, also showing an anti-inflammatory effect [36].

It is known that the homeostasis of human bone metabolism is achieved through a dynamic balance between bone formation by osteoblasts and bone resorption mediated by osteoclasts. Any change in this balance, bone microstructure is destroyed, which leads to increased bone fragility, a process that involves redox imbalance, excessive inflammation, and cell death [8].

At the bone cell level, oxidative stress activates the differentiation of preosteoclasts into osteoclasts, favoring bone resorption. Meanwhile, ROS blocks the activity of osteoblasts and causes apoptosis of osteocytes and osteoblasts, thus reducing osteogenesis, which will cause an increase in turnover for bone remodeling and a decrease in bone mass. Blocking the activity of osteoblasts by oxidative stress, the production of OPG decreases, which will favor the binding of RANKL to RANK and the differentiation of preosteoclasts into mature osteoclasts responsible for the osteolysis process [37].

Oxidative stress and high bone turnover accelerate the formation and accumulation of advanced glycation end products. During osteolysis, osteoclasts secrete collagenases such as matrix metalloproteases (MMP) and cathepsin K (CatK), which degrade type I collagen, leading to high levels of hydroxyproline [38].

Reactive oxygen species produced in excess exert deleterious effects on proteins, lipids, and deoxyribonucleic acid (DNA) in cells, ultimately leading to cellular damage and cell death [39,40]. The use of plant extracts with a content of bioactive compounds with antioxidant properties represents an important interest for health, including bone healing [41,42].

In our study, the SOD and CAT activities increased after EA treatment compared to JR, CF, and CMC, while only the JR extract significantly diminished the MDA production in parallel with low levels of RANKL secretion. JR extract did not influence the secretion of proinflammatory mediators and did not improve significantly the hydroxyproline levels.

*Juglans regia* currently attracts a considerable interest in the biomedical field due to its phytochemical active compounds with pharmacological activities such as antioxidant, anti-inflammatory, and antibacterial activities, with applications in various areas including hepato-renal protection, diabetes, cardioprotective, neuroprotective, and anticancer [43]. Various walnut types and products, such as walnut extract, walnut oil, walnut-derived peptide, and walnut protein hydrolysate, have been evaluated for their properties to activate the Nrf2 signaling pathway, scavenge free radicals, and modify pro-inflammatory pathways, with regulatory effects on chronic diseases (hyperlipidemia, diabetes, arthritis, hypertension, ischemic stroke, Alzheimer’s, seizures, and cancer) [44,45]. In our study, the anti-inflammatory effect was reduced but insignificantly compared to the vehicle, probably due to low concentration at the lesion site or prolonged release in small doses.

In the in vitro models of inflammation, JR male flower extract has shown protective effect against UVB-induced photodamage through antioxidant and anti-inflammatory activities by restoring the SOD, CAT, and glutathione peroxidase (GPx) activities and inhibiting the TNF-α, IL-6, cyclooxygenaze-2 (COX-2), and NF-κB expressions [46].

In a study performed on postmenopausal women, Azizieh et al. demonstrated that women who showed increased serum values of OPG also presented an increase in bone mineral density (BMD), meanwhile women who showed an increase in the ratio of RANKL/OPG presented a decrease in BMD. The same study reported low values of SOD2, peroxiredoxin (PRX), and catalase in postmenopausal females who showed decreases in BMD, but without being able to correlate the serum levels of OPG, RANKL, and markers of oxidative stress with BMD, suggesting a multifactorial cause of bone loss [47].

EA, an important active compound found in considerable concentration in *Juglans regia*, was also widely assessed in various studies. The anti-inflammatory activity of ellagic acid is exerted through up-regulation or down-regulation of several biomarkers by some mechanisms, such as inhibiting store-operated calcium entry-mediated calcium influx, thus decreasing the pro-inflammatory mediators [48], antioxidant and anti-inflammatory mechanisms by reduction of NO, MDA, IL-1β, TNF-α, COX-2, and NF-κB expression, induction of reduced glutathione (GSH) and IL-10 production and substantial boost of SOD and CAT levels [49,50,51,52,53,54,55], inhibition of TNFα-induced inflammation in Caco2 cells, TNFα-induced oxidative stress, and TNFα-induced alterations in redox signaling [56].

In our study, the group treated with EA showed low levels of TNF-α and IL-6 compared to control but higher than values obtained after CF treatment. In the management of osteoarthritis, EA (0, 12.5, 25, and 50 μM, 24 h; 50 mg/kg/day) decreased the production of some inflammatory mediators (NO, prostaglandin E2—PGE2, IL-6, TNF-α, COX-2, inducible form of nitric oxide synthase—iNOS, NF-κB), while up-regulating the expression of collagen type II and aggrecan in IL-1β-induced human chondrocytes in a dose-dependent manner, and higher levels of IL-10 in rats with osteoarthritis induced by monosodium iodoacetate [57,58].

The increase in RANKL expression increased the number of osteoclasts, which favors osteolysis. The RANKL-RANK signaling pathway contributes to the occurrence of osteoclast-related diseases. The interaction between the two proteins, RANKL and RANK, is considered an important therapeutic target and therefore a possible solution in these pathologies. In our study, RANKL expression in the EA group was higher than in the other groups, while in the JR and CF groups it decreased.

Wardhana et al. found significant decreased expression of RANKL in the EA group [59]. Considering the inhibitory effects of EA on RANKL, Xu et al. suggested that EA could directly bind RANK and/or RANKL, explaining the weak expression of RANKL in EA treatments [11]. The effects of EA treatment on bone tissue were also demonstrated by Lin et al., who found that EA significantly repressed osteoclastogenesis through the inhibition by EA of several key transcriptional factors, including NF-κB. EA also inhibits osteoclast function, such as hydroxyapatite resorption [60].

It was demonstrated that JRL extract had a positive effect on BMSC osteogenesis, favoring osteoblast differentiation through BMP2/Smad/Runx2 and Wnt/β-catenin signaling pathways [8]. Kong et al., using a complex extract of JR, performed assays to evaluate the inhibitory effect on RANKL-induced differentiation of osteoclasts from bone marrow-derived macrophages. The results demonstrated that the complex extract of JR can have a role in the treatment of osteolytic diseases such as osteoporosis [61].

Plasma levels of hydroxyproline decrease with age, causing increases in insoluble collagen in the tissues of the elderly. With the help of serum hydroxyproline, important information can be obtained for several processes, such as collagen turnover and bone resorption. At the plasma level, hydroxyproline binds to serum albumin, which plays an important role in the transport and metabolism of hydroxyproline [62]. For our study, the serum levels of hydroxyproline among the study groups had no significant modifications compared to the control group, with its concentration in the EA group being the lowest.

In our study, the calcium level increased in the group treated with EA, while phosphorus (as inorganic or organic compounds) was accumulated in the walnut extract group.

The chemical constitution of the bone tissue is represented by organic components, in particular type I collagen (80%), and inorganic substances, mainly hydroxyapatite, where the Ca/P ratio is 1.67 [63,64]. The ossification process involves the secretion of type I collagen fibers by osteoblasts, which leads to the formation of soft callus, which is amorphous and non-crystalline; in a later stage, the deposition of hydroxyapatites occurs on type I collagen, which constitutes the biomineralization process of the bone and causes the appearance of hard callus [64].

In the specialized literature, there are few data that refer to SEM analyses of bone defects in rats. Suvorova et al. described in a related study on human bone tissue the changes that appeared at SEM on osteoporotic bone compared to healthy bone, in which the number of thick organic fibers increases while bone minerals decrease, causing bone demineralization [65].

Our data demonstrated two primary regenerative pathways involved in the applied therapy. While EA led to the accumulation of calcium-oxygen compounds, a pattern also observed with CF, the plant extract promoted phosphorus deposits in the lesional area. Bone regeneration is generally associated with calcium accumulation, as calcium is a crucial component of the bone matrix, working alongside phosphorus to form hydroxyapatite—the main mineral responsible for bone strength and density. However, in certain conditions, phosphorus accumulation becomes more pronounced than calcium, impacting bone health and regeneration.

One significant clinical correlation is the phosphorus-fluoride interaction, where high fluoride exposure increases phosphorus retention in bone while disrupting calcium metabolism. This imbalance can lead to abnormal bone growth, known as skeletal fluorosis, where bones become hardened but brittle, impairing healthy regeneration (Amaechi and Van Loveren, 2013) [66]. Some studies suggest that phosphate-fluoride complexes, such as fluorapatite (a variant of hydroxyapatite where fluoride replaces hydroxyl ions), may increase bone density to some extent. However, bone quality often suffers, as this disrupts the normal balance of calcium in bone, leading to weaker bones, reduced calcium incorporation, and impaired regeneration, particularly in cases of chronic phosphate accumulation (Riedel et al. [67]). Previous research has shown [6] that the molecular markers observed in our study are indicative of cellular changes consistent with the proposed mechanism. We recognize the importance of validating these findings at the cellular level and suggest this as a valuable direction for future studies to build on our current findings.

Our data positioned the extract as a bone density-increasing agent, which could be useful in conditions where enhancing bone mass is critical, such as osteoporosis or fracture healing. In contrast, EA and CF acted similarly by promoting calcium accumulation in the lesioned cortical bone areas, aligning with findings by Botez et al. [68] and Lin et al. [60], who also reported comparable results.

## 5. Conclusions

The phytochemical composition of *Juglans regia* L. extract was assessed, as well as the total polyphenolic content, flavonoid content, and antioxidant activity. JR extract demonstrated relevant anti-oxidant effect and bone protective activity. CF had anti-inflammatory action and reduced RANKL expression without improvement of antioxidant activity. According to the SEM images, the treatment with EA and JR extract generated the accumulation of Ca and P. Further investigations will be performed in order to explore the influence of both *Juglans regia* L. extract and EA on several biomarkers from bone-related diseases.

## Figures and Tables

**Table 1:** Identification and quantification of several phenolic compounds from the JR extract.

	Compound	P4 Extract (µg/g d.w.) *
1.	(+)-Catechin	322.01 ± 1.49
2.	Caffeic acid	878.15 ± 1.50
3.	Cinnamic acid	0.50 ± 0.02
4.	Ellagic acid	0.34 ± 0.02
5.	Ferulic acid	218.55 ± 1.42
6.	Gallic acid	23.02 ± 0.11
7.	Quercetin	328.14 ± 1.39
8.	Resveratrol	56.92 ± 1.64
9.	Rutin	61.62 ± 0.53
10.	Syringic acid	52.88 ± 0.75

* The results are expressed as mean ± standard deviation for triplicate analysis (n = 3).

## Data Availability

Data are contained within the article.
